# Exploring othering and perceived harmful drinking contexts among risky drinkers: An arts‐based focus group study

**DOI:** 10.1111/bjhp.70008

**Published:** 2025-07-24

**Authors:** Melissa Oldham, Jiexi Yang, Tosan Okpako, Dimitra Kale, James Morris, Claire Garnett, Sara Wallhed Finn, Felix Naughton, Jamie Brown

**Affiliations:** ^1^ Department of Behavioural Science and Health University College London London UK; ^2^ Centre for Addictive Behaviours Research London South Bank University London UK; ^3^ School of Psychological Science University of Bristol Bristol UK; ^4^ Department for Global Public Health Karolinska Institute Solna Sweden; ^5^ School of Health Sciences University of East Anglia Norwich UK

**Keywords:** alcohol, drinking contexts, harmful drinking, othering, risky drinking

## Abstract

**Objectives:**

To explore whether people ‘other’ when making judgements about ‘harmful’ drinking and the drinking contexts (e.g., pub with friends) and features of drinking contexts (e.g., location, company) perceived as being indicative of harmful drinking.

**Design:**

Focus group design with arts‐based methods.

**Methods:**

Risky drinkers (AUDIT‐C ≥ 5; *n* = 20) in four focus groups, drew and discussed contexts and features of contexts they thought indicated harmful drinking. Reflexive thematic analysis was conducted alongside content analysis of drawings informed by themes and prior research.

**Findings:**

There are three deductive themes. The first theme semblance of control referred to people's tendency to differentiate their own drinking practices as controlled and safe in relation to the out‐of‐control behaviours of a harmful drinker. This was seen in the content analysis of drawings where participants tended to draw drinking contexts which were different to those they drank in. The drinking practices perceived as being indicative of harmful drinking were further explored in the second theme harmful drinking contexts. This theme was made up of four subthemes: mental harms, physical harms, social harms and societal harms which were discussed as being differentially associated with different types of drinking contexts. The final theme, features which make drinking ‘harmful’, focused on the features of drinking contexts which participants felt were more indicative of harm. There were seven subthemes: alone, home, amount, drink type, having responsibilities, reason for drinking and timing/pattern.

**Conclusions:**

Drinking contexts fed into the construction of othering narratives among risky drinkers. This has implications for alcohol harm reduction campaigns.

**Statement of Contribution**

**What is already known on this subject?**

- Risky drinkers underestimate the risk of harm; only a minority are motivated to reduce drinking.
- Low problem recognition linked to othering, using an extreme stereotype to construct positive identity.
- Drinking is socially engrained, but no research has explored othering and drinking contexts.

**What does this study add?**

- This study suggests that othering narratives are constructed in relation to contextual information.
- Participants discussed contexts and features of contexts they felt indicated that drinking was harmful.
- This could inform interventions aiming to increase reduction attempts by risky drinkers.

## INTRODUCTION

Globally, alcohol is a leading risk factor for death and disability (Griswold et al., 2018). In a survey of 21 countries, only around a third of adults reported wanting to reduce their alcohol consumption (Davies et al., 2017). This is consistent with the UK, where only around a third of risky drinkers [defined as Alcohol‐Use Disorder Identification Test – Consumption (AUDIT‐C) ≥5 (Bradley et al., 2007) which covers a broad spectrum of drinking] attempted to reduce their drinking in the past year (Alcohol Toolkit Study, 2023). This means that many people who could benefit from reducing their alcohol consumption in the United Kingdom are not currently attempting to do so. Low problem recognition may explain these low rates of motivation to reduce risky drinking. Risky drinkers often underestimate how much they drink relative to other drinkers (Garnett et al., 2015) and their risks of experiencing alcohol‐related harms (Morris et al., 2020). This low problem recognition is related to othering (Alcohol Toolkit Study, 2023). Othering is the process of using an extreme stereotype of a problematic other to construct a relatively positive self‐identity (Khadjesari et al., 2019; Morris et al., 2020). Those who do not perceive their drinking as harmful may engage in othering when confronted with information or feedback on their drinking (Morris et al., 2021). Othering, alongside avoidance or rejection of personally relevant information about health risks (Morris et al., 2024), could lead to the maintenance of low problem recognition (Morris et al., 2020; Morris et al., 2024).

Several theories highlight the importance of beliefs about vulnerability to harm in determining intentions and behaviour. The Health Belief Model (Rosenstock et al., 1988) predicts that an individual's perception of personal susceptibility to risk predicts behaviour change. The Extended Parallel Process Model (Witte, 1994) stresses the role of susceptibility to threats alongside perceived self‐efficacy in determining behaviour change. As such, low problem recognition may partly explain (Melia et al., 2021; Morris et al., 2022; Rosenstock et al., 1988) why the majority of risky drinkers in the United Kingdom (Alcohol Toolkit Study, 2023) and drinkers in a number of countries (Davies et al., 2017) are not motivated to reduce their alcohol consumption. Finding ways to increase reduction attempts among risky drinkers is a public health priority.

Social Practice Theories highlight the importance of understanding drinking as one constituent part of wider social practices (Warde, 2005). Social practices encompass multiple interlinked behaviours and individual and group motivations or ‘meanings’ (Meier et al., 2018; Mielewczyk & Willig, 2007). Through this lens what could be interpreted as one behaviour, such as drinking a beer, can take on very different ‘meanings’ in different contexts (e.g., bonding with friends in a pub or unwinding at home after a hard day). Drinkers in the UK drink in a range of contexts (Ally et al., 2016; Holmes et al., 2024) including ‘at home alone’ and ‘big nights out with friends’. A recent meta‐synthesis of othering explored the characteristics of the ‘problematic other’ constructed by risky drinkers and found that contextual features such as timing and drink type were among those used to differentiate own drinking as non‐harmful (Morris et al., Under Review). It is possible that heavier drinkers create othering narratives about the nature of harmful drinking contexts, including physical, mental or social harms for the drinker or others.

This othering could occur in different ways. There may be drinking contexts that are widely perceived as indicating harmful drinking. For example, lone home drinking (Cook et al., 2023) and drinking in public places such as park benches or in the street (Wallhed Finn et al., 2014) have been discussed as being indicative of harmful drinking. It is likely there are also multiple conflicting narratives about what indicates harmful drinking, depending on the characteristics of the perceiver and the types of contexts they drink in. For example, among a sample of middle‐aged, male drinkers, harmful drinking took the form of a young, inexperienced drinker, drinking to get drunk quickly and causing a public nuisance (Parke et al., 2018). Whereas younger drinkers thought harmful drinking was characterized by older adults habitually consuming bottles of wine daily (Khadjesari et al., 2019).

Little research to date has set out to explicitly explore the link between othering and drinking contexts. As such, we conducted focus groups to explore drinking contexts and features of drinking contexts perceived as being indicators of harmful drinking, and how othering shapes these perceptions. Dual‐process models such as the Reflective‐Impulsive Model (Deutsch & Strack, 2020) draw a distinction between conscious cognitive processing and less reflective heuristic processing. Perceptions on what constitutes harmful drinking may not be something participants have reflected on consciously or critically (Morris et al., 2021). To facilitate reflection, arts‐based methods, such as drawing, can help participants elicit perceptions and beliefs that may be less easily expressed through words (Fraser & al Sayah, 2011; King & Horrocks, 2019; Okpako et al., 2024).

This study addresses the following aims among risky adult drinkers in England

1. Investigate the extent to which people other when making judgements about contexts and features of drinking contexts linked to harmful drinking.
2. Explore which drinking contexts (e.g., drinking at home with partner, in the pub with friends) and specific features of drinking contexts (e.g., location, company) are perceived as being linked to or indicative of harmful drinking.

## MATERIALS AND METHODS

The study was designed in line with guidance recommending holding 3–6 focus groups lasting 1.5 h, each with 6–8 participants and two facilitators (Onwuegbuzie et al., 2009; Plummer d‐Amato, 2008). The Consolidated Criteria for Reporting Qualitative Research (COREQ) checklist (Tong et al., 2007) is included in Appendix S1.

### Design

Focus groups.

### Sample

We held four in‐person focus groups in London between May–July 2024 with 20 adult risky drinkers (AUDIT‐C score ≥5 (Bradley et al., 2007)). Data and thematic saturation were met; no new themes or significant changes to the meanings of codes were identified in the final group.

To reach a broad range of participants, we recruited from multiple sources. This included a database of participants, diverse in age, gender and socioeconomic position, who had previously taken part in studies at University College London (UCL), focused on alcohol and smoking. We also recruited via formal social media advertising (Instagram), posts on the researchers own social media (e.g., Instagram, Weibo) and physical posters in the local area.

We purposively sampled participants, inviting participants to focus groups who had a range of demographic (age, gender, ethnicity and subjective financial situation) and drinking characteristics (full range of AUDIT‐C scores between 5 and 12).

To examine othering and to reduce the likelihood of conflict or participant distress (e.g., if one participant drew or described a context which echoed the drinking contexts of other people in the group), the four focus groups were organized around self‐reported drinking contexts. The four groups were made up of people who reported: lone drinking, drinking with a partner, social drinking in a home and social drinking out of the home.

Each participant was reimbursed with £20 in Amazon vouchers.

### Procedure

After consenting online, participants were screened based on the AUDIT‐C (Bradley et al., 2007). Eligible participants were then asked to provide their name, email, mobile phone number, age, gender, ethnicity and subjective financial situation. Participants also reported the type of contexts they typically drank in. Response options were informed by a recent typology of the most prevalent forms of drinking occasion in the United Kingdom and previous work by the authors (Holmes et al., 2022; Stevely et al., 2024). See pre‐registered protocol for detail (https://osf.io/vs8yb).

Eligible participants were contacted by email to schedule attendance. Focus groups were split into five phases (see topic guide, Appendix S3):

First, MO established guidelines for conversation and confidentiality. Participants introduced themselves and described the last time they had an alcoholic drink. Next, participants were asked to draw what they thought a harmful drinking context looked like. Participants were prompted to think about the location, other behaviours, timing and company. See Appendix S2 for reflexivity statements and reflections on implementing this task. Participants were asked to explain their drawings, highlighting the features that indicated a context was harmful. Then, the facilitator asked questions focused on eliciting participants' broader beliefs about what harmful drinking contexts looked like. As a group, participants were then asked to sort different cards featuring contexts or features of drinking contexts from being more to less harmful. Each context or feature was discussed by the group, allowing space for disagreements and was then placed where most participants felt it should go. Finally, participants were debriefed.

#### Topic guide development

The research team developed a topic guide with reference to prior research (Krueger, 1998; Krueger & Casey, 2014). The topic guide was reviewed by a Patient and Public Involvement (PPI) group to ensure that the questions and tasks were clearly articulated in advance of the focus groups. The PPI group suggested that ‘harmful or problematic drinking’ was the best terminology to use to help participants understand what we were interested in, we use the shorthand of harmful drinking throughout this manuscript. The group also had some helpful suggestions to facilitate trust and richer discussions (e.g., facilitators talking about their own relationship with alcohol via the icebreaker to break down perceived boundaries and making it clear from the start that people did not have to answer questions with reference to their own drinking if they did not want to).

### Analysis

Sessions were audio‐recorded and transcribed verbatim. Drawings were retained and scanned. Recordings were automatically transcribed using Microsoft Office 365 and manually checked and corrected by MO and JY to increase familiarization with the data. Names were replaced with pseudonyms on transcripts. Identifiable information was removed. Data were initially coded in NVivo.

Data were analysed using reflexive thematic analysis underpinned by critical realism. The critical realist perspective is that although reality exists independent of human perception, it is understood through the lens of human experience and interpretation. As such, themes derived through thematic analysis represent patterns based on participants accounts of their experiences. We set out to identify views and experiences around the three areas outlined in the objectives (othering, harmful contexts and harmful features); we deductively coded around these aims. Thematic analysis was conducted in six phases, as outlined by Braun and Clarke (Braun & Clarke, 2006): data familiarization, generating codes, searching for themes, reviewing themes, defining and naming themes and write‐up.

Initially, one transcript was coded by two researchers (MO and JY), with reference to field notes made after each focus group. Following a discussion of the codes, all transcripts were coded by MO and initial themes developed. MO and TO, and then MO, JB and FN met to discuss the themes. The aims of these discussions were twofold. First, to ensure that the research team broadly agreed on the interpretation of data and to limit (but not eliminate) researcher bias. Second, discussions prompted reflection on how themes were arrived at and interactions between themes.

Content analysis of the different types of contexts (e.g., big night out) included in the sketches was undertaken by MO and discussed with TO for verification. Drawings were coded as to which of the nine types of drinking contexts used in the screening survey they most resembled. Most of the drawings fit within this framework clearly; however, two participants drew examples of street drinking, so these were coded inductively. When undertaking this content analysis of images, MO referred to participant descriptions of their drawings to ensure no important features of contexts described by participants were missed or misinterpreted (e.g., clarifying whether a scene was a party in a house or a social event in a bar).

As well as informing the content analysis, the discussions which took place around each of the drawings (e.g., participants explaining their pictures or participants commenting on or making comparisons with other drawings) were also coded as part of the thematic analysis.

## RESULTS

Three hundred and thirty‐eight people started the screening survey; 164 people were screened into the study. Of these, 69 were invited to attend a focus group. Given purposive sampling on demographic and drinking characteristics, not all eligible participants were invited to a group. Twenty participants attended four focus groups and were diverse in terms of gender, age, ethnicity and subjective financial status (Table 1).

**TABLE 1:** Participant characteristics overall and by group.

	FG1 drinking alone (*n* = 5)	FG2 social out of home (*n* = 5)	FG3 with partner (*n* = 5)	FG4 social in home (*n* = 5)	Total (*n* = 20)
Age					
18–30	5	2	2	2	11
30–59	0	1	1	2	4
60+	0	2	2	1	5
Gender					
Man	3	4	2	2	11
Woman	2	1	2	3	8
Non‐Binary	0	0	1	0	1
Ethnicity					
Asian	3	0	0	1	4
Black	0	0	1	0	1
Mixed	0	1	1	0	2
White	1	4	2	4	11
Other	1	0	1	0	2
Subjective financial situation					
Live comfortably	1	3	2	0	6
Meet needs with a little left	2	1	2	1	6
Just meet basic expectations	2	1	0	4	7
Don't meet basic expectations	0	0	1	0	1

Abbreviation: FG, Focus group.

Three deductive themes were generated, reflecting the study aims of othering, harmful contexts and harmful features. The first theme, *semblance of control*, refers to participants tendency to differentiate their own drinking practices as controlled and safe in relation to the out‐of‐control behaviours of a harmful drinker. The drinking practices perceived as being indicative of harmful drinking are further explored in the second theme, *harmful drinking contexts*. This theme is made up of four subthemes: *mental harms, physical harms, social harms* and *societal harms*, which are differentially associated with different types of drinking contexts. The final theme, *features which make drinking ‘harmful’*, focuses on the features of drinking contexts which participants felt were more indicative of harm. There are seven subthemes: *alone, home, amount, drink type, having responsibilities, reason for drinking and timing/pattern*. Themes, subthemes and codes are outlined in Table S1 and discussed below.

### Othering in depictions and descriptions of harmful drinking contexts

When asked to draw a harmful drinking context, most participants drew someone drinking alone at home or as part of a big day or night out with friends. Both were perceived as being heavy drinking occasions (See Table 2).

**TABLE 2:** Content analysis of drawings.

Contexts	FG1—Drinking alone	FG2—Social out of home	FG3—With partner	FG4—Social in home	Total
Alone at home	0	3	4	3	10
Big day or night out	0	0	2	2	4
Street drinking^a^	1	1	0	0	2
With partner or family at home	1	0	0	0	1
Social event in a home	1	0	0	0	1
Pub with friends	0	0	0	1	1
Work drinks	1	0	0	0	1
Pub alone	0	0	0	0	0
Meal out	0	0	0	0	0
Out with Partner	0	0	0	0	0

*Note*: Some participants drew more than one scene.

Abbreviation: FG, focus group.

^a^ Coded as covered in previous research on harmful drinking contexts (Wallhed Finn et al., 2014).

**FIGURE 1:**
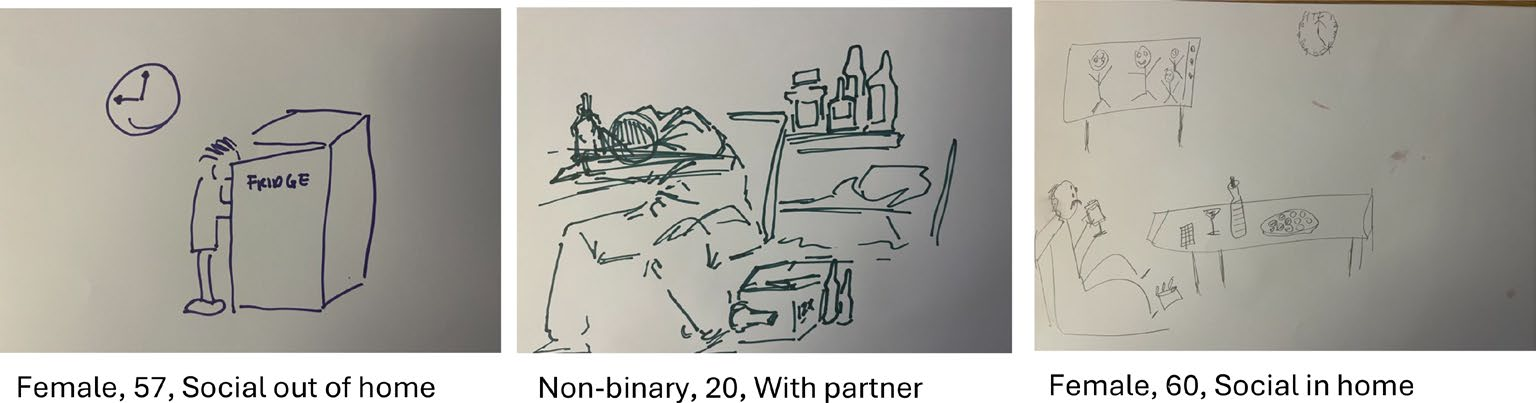
Participants drawings representing lone drinking at home.

Participants drawings showed evidence of othering. For example, in Focus Groups 2–4, most participants depicted somebody drinking alone at home, see Figure 1. Home alone was perceived as being associated with drinking to cope.

In these focus groups, when asked how they would change images to be indicative of less harmful drinking, most said they would add people.

However, in the focus group made up of participants who reported lone drinking, no respondents drew someone drinking alone at home, though one participant did draw a lone street drinker.

**FIGURE 2:**
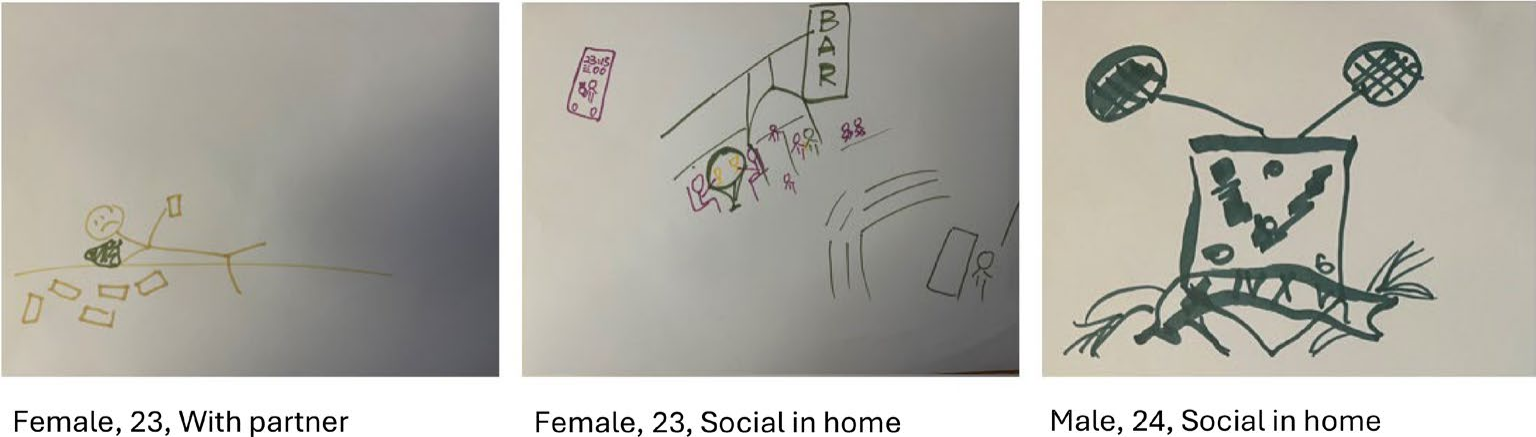
Participants drawings representing big days or nights out.

Big days and nights out were seen as being associated with excessive drinking and harm to the drinker, as well as potential harms to others through violence or victimization (Figure 2). The first image represents somebody who has collapsed on a night out with friends. The second image was described as a busy bar with a higher likelihood of sexual violence. Whilst the third image depicts a University Sports Society emblem, representing excessive and harmful drinking cultures that characterize participation in university sport societies. No participants in the focus group made up of social drinkers out of the home drew a big day or night out.

There was also evidence of othering in the thematic analysis. This is encompassed by the theme *semblance of control*. Participants spoke about harmful drinking as operating outside the bounds of ‘normal’ drinking. Participants described ‘red flags’ which indicated harmful drinking, like a drink type which was ‘inappropriate’ to the setting, such as ordering shots or drinking spirits at lunchtime.

That was always my red flag, if I saw someone like drinking like spirits at an unacceptable time. But then I'm hypocrite because I also have a gin and tonic at lunchtime sometimes. Female, 43, With partner

In line with this, many participants talked about having personal rules to guide their own drinking practices. These often mapped on to the features of drinking contexts discussed below that determined whether drinking contexts were perceived as being harmful, particularly around *reason for drinking* and *timing/patterns*.

It's just a personal thing, but like on a weekday, I would never drink before 8 and I and it's a weird, stupid thing, but I only drink from Thursday onwards and then come Monday, that's it. I stop. So to me, it's very time specific. Female, 43, With partner

Most of the time when I feel stressed, I wouldn't really drink. Because I feel myself, stress is having a problem, so I just want to solve the problem not to avoid it using alcohol. Female, 26, Drinking alone

These personal rules seemed to serve the purpose of reassuring participants, and perhaps others, that they were in control of their drinking. This was frequently contrasted with a stereotypical, out‐of‐control harmful drinking other, who has gone beyond ‘normal’ drinking.

I think for me to say problematic, it's like if you physically cannot live your life and kind of almost stick to like society's rules, like you don't get up because you're drunk and you don't go to work because you've got to have a drink… then I guess you're an alcoholic. Female, 43, With partner

In line with this separation, fewer participants depicted ‘street drinking’ than may have been expected, given previous literature where this is discussed as being commonly perceived as a harmful drinking stereotype. Throughout discussions this seemed to be because participants constructed a hierarchy of harms, and street drinking was seen as being on a different level of harmful drinking which could not be compared to people who were ‘functioning’.

That particular example was kind of like in another dimension, because we are like talking about like the people that are like middle class or you know have like normal jobs… But this example is like from a person that has, like really big underlying financial issues, like health issues, family issues, maybe doesn't have job or a home to live… we cannot compare him to the other situations. Male, 28, Drinking alone

Below the contexts and features of contexts perceived as being indicative of more harmful drinking are explored in more detail.

### Contexts linked with harmful drinking

The second theme, *harmful drinking contexts*, encompasses four subthemes: *physical harms*, *mental harms, social harms* and *societal harms*.

*Physical harms* were one of the most discussed themes. Participants mostly focused on short‐term harms (e.g., vomit, hangovers) or victimization (e.g., violence, sexual violence). These harms tended to be associated with social drinking out of the home.

I think pubs, pubs, outside pubs late at night, that's usually where most of the trouble is, especially around here. Male, 25, Drinking alone

Some participants, particularly those who were older, talked about more chronic health harms of alcohol such as fatty liver disease and cancers. These harms were discussed mostly in relation to lone or home drinking. This may reflect the themes expanded on below that participants tended to link lone home drinking with frequent and excessive drinking and drinking to cope.

I think yes, [drinking habitually at home with your partner] is fine with your, like, social life and your daily function, but not for your liver. Male, 28, Drinking alone

*Mental harms* were not discussed as much as *physical harms* and were discussed mostly in the context of lone home drinking; again, this was linked with *timing/pattern* and *reasons for drinking*, which are expanded on below.

You're a bit depressed, it's like 3:00 pm on a Monday, so you open a bottle of vodka or gin, and that's at home and I think that's probably quite problematic. Female, 43, With partner

I also think it's a question of mental well‐being, if I have, you know, 12 pints, let's say out with friends. I will probably come away from that feeling much better than if I was drinking the same at home. Male, 27, Social out of home

*Social harms* were applicable across a range of contexts. When discussing rowdy behaviour or bothering others, this tended to focus on social occasions with big groups, such as parties or big days or nights out. Participants described patterns of antisocial behaviour clustered in areas with a high density of bars and clubs and on public transport on weekend evenings.

I was coming back from a movie on the Tube and there were a bunch of people around 10‐15 people, I guess they were coming from a pub drunk… and some other lady was sitting at the other corner. And they all came all together, and they were all over the couch, and she was quite on edge. Male, 26, Drinking alone

Another facet to *social harms*, was damage to relationships and reputation. Interestingly this was associated particularly with drinking in the home. Participants felt that drinking in public contexts was likely to inhibit behaviour and reduce the likelihood of arguing with partners, given social norms around acceptable behaviour. Participants felt that the risk of relationship conflict were higher in a home setting. This is expanded upon below in the ‘Home’ theme.

Interaction between participants:

Female, 43, With partner It's easier to lose your temper with somebody when you're at home. If you are out, you often check yourself, don't you? You don't want people thinking oh, my God, that couple there, they are going at it. How awkward.

Male, 62, With partner Yeah, yeah. You sort of, you know, you, you regulate, don't you outside?

Social harms were also discussed in relation to work drinks, given the perceived high stakes environment and potential damage to professional reputation.

Interaction between participants:

Male, 26, Drinking alone It depends upon the kind of people you're out with. [murmured agreement] If there are some less friendly team in the workplace and you say something and they eventually..

Female, 23, Drinking alone in response You might start saying things that you shouldn't say to your work colleagues, yeah.

Male, 25, Drinking alone in response I think the potential for harm when you're at work drinks is higher than say with friends around you. There's more chance of getting things wrong. On the flip side, I think you're also on best behaviour a bit, right?

Finally, participants also discussed *societal harms*, which they felt were bound up with British drinking culture, and its perceived tendency towards excessive consumption. Participants focused on the proliferation of alcohol in social spaces; this was mostly discussed in the context of work drinks. Participants felt that alcohol was central to networking and socializing opportunities across most careers and that this was harmful as it perpetuated the normalization of alcohol. This was also seen as being exclusionary to people who did not drink for religious or other reasons which represented a societal threat to multiculturalism.

I feel like I have lots of friends that don't drink a lot or at all… They're like, even if we do go to an activity, a lot of the time people will go to the pub afterward, and they can't join in. Female, 23, With partner

It can be exclusionary. Like, if you don't drink or your religion won't allow you to drink, and you're kind of forced to go to work drinks, then that's a massive problem. Non‐Binary, 20, With partner

### Features of drinking contexts linked with harmful drinking

The final theme focused on features which make drinking ‘harmful’. The seven subthemes are: alone, home, amount, drink type, having responsibilities, reason for drinking, and timing/pattern.

#### Alone

Drinking *alone*, particularly at *home*, was one of the most cited indicators that drinking was harmful. This seemed to be linked closely with the *reason for drinking*. Most participants believed that drinking alone indicated somebody was isolated and drinking to numb feelings of stress or sadness. This was also related to the *amount*, as people drinking to ‘numb’ themselves were perceived as being likely to drink heavily. Participants described drinking alone as being a step along a ‘slippery slope’ that led to social isolation and heavier drinking. The use of ‘slippery slope’ denotes an at first gradual but quickly spiralling loss of control. It invokes the idea that some drinkers are on the precipice of losing control and places the emphasis on the drinker to navigate drinking patterns to maintain control.

Interaction between participants:

Female, 23, With partner I think for me, when I go to pubs and I see an older man by themselves, I see that more often than anything else. I think… I mean I obviously don't say anything but its kind of like, I just think… I don't know how to say it properly.

Male, 62, With partner in response Does that depress you? Seeing older men on their own?

Female, 23, With partner, in response Well, I think wouldn't they like to be there with someone else instead? I'm sure they would prefer that than being by themselves.

Conversely, most participants endorsed the idea that ‘happy’ or ‘social’ drinking was less harmful and had mental or social benefits that balanced out the physical harmful aspects of drinking.

Social drinking is less harmful than drinking alone in your home, because well, first of all, social drinks cannot happen every night… And second is that when you're socialising, talking with your friends and well, it is, it's a better context than, you know, like sitting alone in your home, drinking and overthinking. Male, 28, Drinking alone

There was some disagreement about the harms of lone drinking. Particularly in the focus group formed of participants who reported drinking alone, participants felt that going to a public space alone to drink could be ‘brave’ and ‘empowering’. The use of ‘brave’ could refer to the idea that drinking alone is outside of normal or acceptable drinking patterns. This intersected with *reason for drinking* and *amount*, if someone was drinking alone lightly or for pleasure, this was not seen as being indicative of harmful drinking.

[If] there is no other people that that you can go out with… if you actually are brave enough to like enjoy yourself alone in a like a pub… I think that's kind of a good context. Male, 26, Drinking alone

#### Home

Participants felt that home drinking was unregulated in ways which increased the risk of harm. As discussed above, people felt that there was less obligation to conform with social norms around behaviour, both in terms of *amount* consumed and treatment of others. Drinking at home, particularly *alone*, was also seen as being harmful because there would be no one to help in case of emergency, and no one to step in and ‘stop serving’. Participants also felt that social events out of the home had a natural stopping point, when the bar or club shut, whereas home drinking could continue indefinitely. These points reference home drinking as being less controlled than in other contexts.

I guess part of the fact that it's also an unregulated place means that there's also no one legally who has to tell you to stop. Like, it's not like a bartender where if you're intoxicated, there's repercussions if they don't stop you, it's at home. Non‐Binary, 20, With partner

The relative ease and cheapness of drinking at home was also given as a reason home drinking might be more harmful, participants felt that more alcohol would be consumed, increasing the risk of harms.

It's the cost, when you're at home, you're not aware of how much you're spending because it's already there. Female, 60, Social drinking in a home

#### Amount

Another of the most cited features of drinking contexts considered to be indicative of harmful drinking was ‘excessive’ consumption. Perceptions of what constituted excessive consumption varied substantially from person to person, and mostly did not map on to UK drinking guideline definitions of low risk drinking.

It depends how much I'm drinking, doesn't it? …Pub alone every day, 1 gin and tonic before I catch the tube to go home or pub alone everyday sit in there until closing time. Yeah, two different things. Male, 65, Social out of home

This was frequently contrasted with ‘drinking responsibly’ and ‘knowing your limits’. ‘Limit’ could be meant in terms of damaging health but seemed to be used more in reference to the point at which they started losing control. In line with the *semblance of control* theme above, most participants felt they did not surpass their own drinking limits and that they were in control of their drinking.

It is important to be responsible when drinking because if the person is not responsible well then like in any setting, to be honest, there's a risk of like turning like a normal setting to like a chaotic setting. Female, 23, Drinking alone

#### Drink type

Participants felt that drinking cheap alcohol was indicative of problem drinking. There was intersection again with the *reason for drinking*, whereby participants felt that drinking cheap alcohol demonstrated that someone was drinking for the numbing effect to relieve stress or unhappiness, as opposed to drinking for pleasure.

Say it's like Lidl own brand vodka that's cheap and cheerful you're not, you're drinking it to get drunk. As opposed to a nice type of vodka or nice gin and tonic that you're kind of having for the taste. Female, 43, With partner

In two focus groups, there was also a discussion of how this was moderated by an individual's social class or economic status. Participants felt that behaviours that would be demonized if carried out by individuals from less advantaged backgrounds, would be seen as socially acceptable if carried out by someone more advantaged, this is demonstrated in the interaction below.

Male, 61, Social out of home If you're a certain, from a certain background, say if you're middle class and upwards…

Female, 57, Social out of home I think I agree with what you said there, it's like if someone goes to his, you know, centuries old members club down [location] and has a stiff gin and tonic at 11:00 they will be thought of very differently from the person who goes into a working man's club and does the same.

Male, 61, Social out of home Or the person that goes to you know that goes to Tesco's and gets…

Female, 57, Social out of home Yeah and sits at home.

#### Reason for drinking

As mentioned above, participants consistently differentiated between ‘good’ and ‘bad’ *reasons for drinking*. Some ‘good’ reasons were to compliment a meal or as part of social events and celebrations, such as weddings or birthdays where alcohol was not seen as being the focus of the occasion, but rather something that enhanced, or positively augmented an already positive experience. This seemed related to the earlier points around *semblance of control*, alcohol was not centred in these occasions, suggesting it was nice to have but not needed.

You've got to experience something like, some celebration with friends or whatever. And so that yeah cancels out some of the other the negatives that might be there in other settings. Male, 33, Social drinking in a home

As described above, conversely ‘bad’ reasons for drinking were to cope or drinking for the medicinal effects of alcohol. Describing alcohol as a medicine suggests an underlying condition which needs to be treated or cured. This could be indicative of a reliance on alcohol or a loss of control. The line between coping and relaxing often seemed blurred, participants generally talked about themselves using alcohol to relax but other people using alcohol to cope. Participants expanded on the differences between the two. People who were drinking to cope were more likely to be home, alone and drinking strong, cheap alcohol quickly. By contrast, drinking to relax was seen as being something done alongside other activities to change your mood, such as seeing friends.

I think with relaxing you're usually with people and you're just, you know, you're not necessarily conscious of drinking, you're busy chatting and you're enjoying yourself. Whereas when you're on your own watching television or you're stressed, you just drink. It's almost like a medicine. Female, 60, Social drinking in a home

I think maybe the quantity and the pace is different between relaxing and coping, I think if you're relaxing, you might savour it and enjoy it and you might only have a couple. But if you're coping, it's like straight up and maybe faster, like chugging or shots or straight liquor. Male, 33, Social drinking in a home

#### Having responsibilities

Participants described a range of scenarios where drinking was seen as being particularly harmful as the individual drinking was ‘on duty’ or ‘responsible’. Drinking in these settings which could negatively harm others was seen as an indicator that an individual was not ‘functioning’. These included people drinking at work, when caring for young children or driving. This is captured in the interaction below:

Male, 25, Drinking alone I noted that your [drawing] was during the daytime and during work. Which was, yeah, obviously not an ideal place to be drinking. Especially with that much responsibility. I mean, it's similar to mine, like if you're in charge of looking after young people… that's probably the worst place to…

Male, 26, Drinking alone in response That emotionally traumatises [points to Male 25's picture depicting a man shouting at his wife and children], this physically traumatises [his own picture depicting someone drink driving whilst driving a school bus].

#### Timing/pattern

One factor seen as underlying all drinking contexts was related to the timing and pattern of occasions. Occasions that were seen as being infrequent, such as celebrations or big days out, were seen as not being as harmful and falling outside of the recommended constraints of more regular occasions. Conversely, developing ‘bad habits’, such as frequently drinking in the same setting or company, such as at home alone or with a partner, was seen as more harmful, and seen to have more of an impact on long‐term health than less frequent, social events.

I think this [big day/night out with friends] you do every now and again but this [drinking at home with partner] you do every day, or more regularly, so I would say it's more harmful because of the quantity that you're consuming, but you don't go to birthdays and weddings all the time. Male, 25, Drinking Alone

Alongside frequency, time of day was seen as being an indicator of harmful drinking contexts. Drinking in the morning was almost universally seen as being a sign that a context was harmful, and some participants indicated that day drinking was associated with more harm. When describing morning or day drinking, most participants referred to people drinking alone at home.

I think people drinking in the mornings. You know I have had a number of friends and some have died from alcohol and you know I noticed they were drinking first thing. That is a bit of a taboo for me. Male, 62, With partner

## DISCUSSION

This study aimed to investigate the extent to which people other when making judgements about contexts and features of drinking contexts linked to harmful drinking and to explore which drinking contexts and specific features of drinking contexts are perceived as being linked to or indicative of harmful drinking. There is some evidence of othering in prior qualitative research (Khadjesari et al., 2019; Parke et al., 2018) but this is the first study which has set out to examine this explicitly, selecting focus groups by the contexts in which people tend to drink alcohol.

There was evidence of othering in both the thematic analysis of data and the content analysis of drawings. In line with other literature (Davies et al., 2018; Morris et al., 2022; Morris et al., Under Review) participants justified their own drinking in relation to a stereotypical problematic other, defined primarily by a lack of control and an inability to function. Drinking contexts were relevant to the construction of this ‘problematic other’ and *semblance of control*. Participants discussed contexts and features of contexts they viewed as harmful or outside the bounds of ‘normal’ drinking. A failure to drink in a ‘normal’ or socially acceptable way was perceived as indicating that someone was drinking harmfully. Participants disclosed personal rules which they used to govern their own drinking, these were often individual, specific and related to features of contexts perceived as being harmful such as *timing/pattern* or *reason for drinking*. These personal rules seemed to serve the purpose of protecting participants self‐identity as non‐problematic drinkers (Morris et al., Under Review) by reassuring participants that they were operating within the bounds of normal, controlled and therefore less harmful drinking. The most drawn harmful contexts were drinking alone at home and big days/nights out. However, in the groups constructed around drinking alone no participants drew someone drinking at home alone and in the social out of home drinking group, no participants drew a big day or night out, suggesting that perceptions of what constitutes a harmful drinking context are associated with an individual's own drinking behaviour. The direction of this relationship is unclear.

The second theme focused on *harmful drinking contexts*. Participants described a range of alcohol‐related harms which were linked with different drinking contexts. *Physical harms* tended to be associated with social drinking out of the home (violence or victimization) or drinking alone at home (long‐term health conditions). *Mental harms* were also linked with drinking alone, particularly at home. *Social harms* were associated with heavier drinking in a range of contexts including social drinking out of the home (violence) and drinking at home (relationship damage) or work drinks (reputational damage). *Societal harms* were discussed mostly in the context of work drinks (normalizing alcohol consumption and exclusionary to modern multicultural Britain). This work builds on the work of previous studies which have outlined that lone drinking, home drinking and street drinking (Cook et al., 2023; Wallhed Finn et al., 2014) are contexts perceived as being harmful. By taking a broader focus in this study, participants discussed different types and gradients of harm which resulted in a wider range of drinking contexts being discussed as harmful. Previous literature and findings from this study also highlight nuances in contexts which are perceived as being harmful. For example, some participants in this study, particularly those who reported lone home drinking but also among some who drew lone drinking at home as their harmful context, felt that drinking lightly in this setting was acceptable when drinking for pleasure (as opposed to coping with sadness/stress). This echoes previous literature in which mothers were excused from drinking lightly alone because they were not able to drink in other contexts (Cook et al., 2023). This also supports dual‐process models such as the Reflective‐Impulsive Model (Deutsch & Strack, 2020), and shows that participants who drew a particular context at the start of the sessions may have a more nuanced view when engaging in discussions or critical reasoning.

The third theme focused on factors which make drinking ‘harmful’. Seven subthemes focused on features of drinking contexts thought of as being indicative of more harmful drinking. These features mapped onto different elements of Social Practice Theory, including Materials (e.g., alcoholic beverages), Meanings (e.g., drinking to cope) and Competencies (e.g., keeping intoxication levels appropriate to context) (Meier et al., 2018). Some of these features, such as *drink type* and *amount*, were captured in a previous meta‐synthesis of othering (Morris et al., Under Review). However, this work builds on previous work by exploring a broader range of factors, the interactions between them and explicitly attempting to draw out othering through the clustering of similar drinkers within focus groups and through questions contrasting others and own drinking. For example, the *reason for drinking* and the extent to which alcohol was centred within a context related to perceptions of harm. People drinking alone at home to self‐medicate against stress or pain were contrasted with contexts where alcohol was consumed for pleasure or was one constituent part of wider social practices such as a wedding.

This study has implications for tackling alcohol harms at the individual and population level. Less than a third of risky drinkers in the United Kingdom made a last year attempt to reduce their drinking (Alcohol Toolkit Study, 2023). Othering has been identified as a key factor in maintaining low problem recognition (Morris et al., 2022), and theories such as the Health Belief model highlight the importance of beliefs about susceptibility to harm in determining behaviour and intentions (Rosenstock et al., 1988). This study suggests that othering narratives are constructed in relation to contextual information. If people ‘other’ and do not see themselves as a harmful drinker, they may be less likely to be motivated to cut down, or if motivated, less likely to seek external help or support. Future research should establish whether othering is linked to lower problem recognition and reduction attempts and whether public health messaging challenging othering narratives leads to increased motivation to reduce consumption and help seeking. This study has further implications for how harmful drinking is framed in wider society. Harmful drinking throughout this study was frequently contrasted with ‘drinking responsibly’ and ‘knowing your limits’, the prevalent use of these phrases, which echo industry narratives emphasizing individual responsibility (Maani Hessari & Petticrew, 2018), demonstrate how industry campaigns can permeate public consciousness and shape perspectives. A recent study summarized the gap between industry, policy, public and expert opinions and developed framing messages aimed at narrowing this gap and moving the focus from one on personal responsibility and clinical treatment towards one of societal responsibility and harm reduction (Fitzgerald et al., 2024).

### Strengths and limitations

A strength of this study was the use of arts‐based approaches, which are participatory, engaging and may facilitate less confident participants to contribute (Cook et al., 2023). Certainly, in this study, the drawing task seemed an effective way to build rapport between participants and encouraged the flow of ideas. Participants commented on each other's drawings, highlighting contexts or features they agreed with and often circled back to features in each other's drawings throughout the rest of the focus group. This was also a simple and effective way to examine othering, as the images and participants descriptions of them differed between focus groups. A further strength was the sample diversity in terms of ethnicity and gender. However, only a minority of participants reported difficulty meeting financial obligations, and none of the population were homeless; just over half of the sample were under 30. Given likely differences in drinking practices among demographic groups, future research should examine potential demographic differences in perceptions of harm. The sessions were designed to have people who reported drinking in similar contexts grouped together. However, whilst everyone assigned to a particular group reported drinking within that context, most participants reported drinking in a range of contexts. We did not ask about the frequency with which they drank in different contexts. As such, someone in the ‘drinking alone’ group might occasionally drink alone but spend more of their time drinking in social settings.

## CONCLUSION

Drinking contexts fed into the construction of othering narratives among risky drinkers. Participants constructed personal rules, often focused on contextual features such as timings or reasons for drinking, which served to differentiate their drinking practices from those of a stereotypical out of control, harmful drinker. When asked to reflect on harmful drinking contexts, risky drinkers in England described a broad spectrum of harms which were differentially applicable to different drinking contexts. Participants most frequently highlighted drinking alone at home, or big days or nights out as more harmful contexts. Participants also described a range of features of contexts which they felt indicated that contexts were harmful such as the reason for drinking, the amount, type and cost of alcohol and patterns of consumption. These findings have implications for future public health campaigns aiming to increase alcohol reduction attempts among risky drinkers.

## AUTHOR CONTRIBUTIONS

**Melissa Oldham:** Conceptualization; data curation; formal analysis; funding acquisition; writing – original draft; writing – review and editing; methodology; investigation; validation. **Jiexi Yang:** Conceptualization; data curation; formal analysis; investigation; methodology; validation; writing – review and editing. **Tosan Okpako:** Conceptualization; investigation; methodology; validation; writing – review and editing. **Dimitra Kale:** Conceptualization; methodology; writing – review and editing. **James Morris:** Conceptualization; methodology; writing – review and editing. **Claire Garnett:** Conceptualization; writing – review and editing; methodology. **Sara Wallhed Finn:** Conceptualization; writing – review and editing; methodology. **Felix Naughton:** Conceptualization; funding acquisition; methodology; supervision; writing – review and editing. **Jamie Brown:** Conceptualization; funding acquisition; methodology; writing – review and editing; supervision.

## Supporting information


Data S1:

## Data Availability

The data that support the findings of this study are openly available in OSF at https://osf.io/3e4by/.
