# Supplementary material for: Exploring othering and perceived harmful drinking contexts among risky drinkers: An arts‐based focus group study
Source: Br J Health Psychol. 2025 Jul 24;30(3):e70008. doi: 10.1111/bjhp.70008 (PMC12287896; doi:10.1111/bjhp.70008)

**Supplementary Materials**

**Appendix 1 – COREQ**

[Uploaded separately]

**Appendix 2 – Topic Guide**

| Phase | Questions and Tasks |
| --- | --- |
| Introduction (10 minutes) | Introduce the facilitators and talk about the type of research interests each has.  Explain aims of the focus group:  “*The goal of the focus group is to understand what could be seen as problematic or harmful drinking. When we say problematic or harmful, this could be in terms of social harms such as being damaging to relationships or losing work, or impacts on physical or mental health. These could be short term or longer term. As part of this you will not be expected to share any personal information about you or your own drinking unless you want to, we are talking more generally about what ‘harmful’ or ‘problem’ drinking can look like.”*  Explain presence of the audio recorders.  Go over consent and what this means. Ask participants if they are still happy to take part.  Go over focus group “ground rules” and confidentiality:  “*All information collected today will be confidential and no one’s name will be disclosed or linked to any quotes in the final report. As this is a group discussion, please respect the privacy of your fellow participants and don’t share the contents of this discussion outside of this room.*  *We all have different views and life experiences and it’s ok if you don’t always agree with other people in the group. Please do feel free to share your thoughts and opinions even if they differ from what other people have said. That being said let’s remember to be respectful to each other when expressing disagreements. We have a limited amount of time so please do not be offended if I stop you to redirect the conversation slightly or to bring someone else in, I just want to make sure there is enough time to get through everything and to hear from everyone. I will start by asking a few questions but really I want most of the session to be a conversation between you all, I will occasionally direct your conversation towards a certain topic.*”.  **Icebreaker**: Can we each introduce ourselves by name and as an icebreaker can you tell us something about the last time you drank alcohol? |
| **Phase 2 – Art Based Task (25 minutes)** | **Drawing Task**  Explain task to group. Hand out markers and card paper:  “*In this part of the focus group we would like you each to think about what you picture when you think of problematic or harmful drinking. Where is the drinking happening? Who is there? What activities or other behaviours are accompanying the drinking? What types of drink are being drunk? What day is it? What time of day is it? Please create a drawing or diagram to represent this.*  *Don’t worry about your artistic ability being judged, the goal of this exercise is to help generate ideas. If anyone isn’t up to drawing, I or one of the other researchers can help you. Alternatively, you can write down your ideas as bullet points or a word cloud on the card. You’ll have around 10 minutes for this activity. After you have each completed a picture we will share them and chat about them a little bit with the group.”*  **Feedback and discussion**   1. Now we will go around the room and share our sketches and ideas. What are the key parts of the setting or context which demonstrate someone is drinking in a problematic or harmful way? Feel free to share your thoughts on each other’s drawings, sharing what you liked or what you would change or add. 2. If I had asked you to draw someone drinking in a non-problematic or less harmful way do you think your drawing would be different, and if so, how? |
| **Discussion (30 minutes)** | 1. Are there key ‘red flags’ which you think demonstrate someone is a problematic or harmful drinker? 2. What about contexts, so this could be drinking at home alone or with a partner or in a pub or restaurant with friends, are there any contexts or settings which would be a red flag to you?   Prompt: Where and when is it okay to drink/where is it not okay? Is this affected at all by seasons or weather?   1. Do you ever drink in the ways you have just described?   Prompts: Do you think you drink in a harmful way? If yes, what makes you think that? If no, why not? Do you drink differently when you are stressed or unhappy?   1. Do you ever feel guilty about drinking alcohol and are there any types of settings or contexts which make you feel more guilty?   Prompt: Are there types of drinking occasion that make you feel less guilty?   1. What are the signs which would make you think someone should try and drink less alcohol?   Prompt: Are there particular settings or drinking contexts that would make you more worried about someone else’s drinking?   1. Do you ever want to try and drink less alcohol? What prompts those thoughts? |
| **Card Sorting Task (20 minutes)** | Participants will be provided with some cards with drinking contexts (e.g. home with partner, out with friends) or features of drinking occasions (e.g. drinking alone). They will be asked to place these cards on a line where “problematic or harmful drinking” is on one end and “non-problematic or less harmful drinking” is on the other. They will also be provided with post it notes to add additional contexts or features which they think are important. |
| **Debrief (5 minutes)** | 1. Does anyone have anything else they would like to share?  2. Does anyone have any questions?  Clarify any misconceptions (if relevant).  Give out any remaining refreshments.  Advise participants that they will be emailed their compensation.  Thank them again for participating. |

**Appendix 3 - Research Team and Reflexivity**

*Personal Characteristics of Interviewers*

There were three facilitators, two in each group. MO is a female Senior Research Fellow at UCL and has a PhD in Health Psychology. She is a mixed-methods researcher and has previously conducted semi-structured interviews and focus groups.

TO is a female PhD student at UCL with an MSc in Public Health and Health Promotion. She is a mixed-methods researcher with previous experience conducting and analysing data from focus groups and has attended training courses in interviewing.

JY is a female master’s student at UCL. She had not previously undertaken focus group research but has read papers on qualitative methods, focus group facilitation and analysis.

*Relationship with Participants*

Participants had not met the researchers before the focus groups but exchanged emails regarding logistics of the focus group. They may have had some knowledge of the interviewers and their roles within UCL through study documentation. Background on the research focus of the two facilitators and the goals of the specific research were explained to participants at the start of the focus groups. That we research alcohol consumption may have indicated to participants that we have opinions on alcohol consumption and drinking practices. We are aware that this could have created a perceived imbalance of power – that participants might have felt pressured to hide how much they drink or the ways in which they drink, or to present a version of themselves which is more concerned about drinking less. We aimed to create a non-judgemental and safe environment in which participants felt able to express their views honestly and openly. To achieve this and guided by the advice of experts by experience, we made the aims of the research clear to participants at the start of the group and clarified that the aim was not to diagnose or make personal evaluations about anyone else’s drinking. Participants were also told that they could share views in the third person if they preferred (e.g. “some people might think”).

The experts by experience we spoke to also suggested that the researchers share information about their own drinking. Through the ice-breaker task, the researchers shared information about their own drinking practices. These tended to be in social settings such as watching football in the pub with friends, or celebrating a birthday. The researchers drinking practices did not seem to influence the types of drinking occasions that the participants described. As these varied with some people reporting social occasions and others reporting drinking at home or alone.

*Arts-based task*

Overall, the arts-based task worked better than we expected. We had expected some participants to be more hostile with this task but clarifying that we weren’t interested in artistic skill and that it was essentially a tool to spark ideas seemed to be acceptable to participants. Some did seem a little embarrassed when presenting their pictures to the group and joked about not being an artist. However, this seemed to break the ice and improve rapport in groups. When asking participants to explain their drawings the level of detail adopted by the first participant set the tone for how much participants shared. Asking more follow-up questions of the first person seemed to help with eliciting more detail on drawings from the rest of the group.

**Appendix 4 – Overview of Coding**

Table S1**.** Themes, subthemes and codes

| **Theme** | **Subtheme** | **Codes** | |
| --- | --- | --- | --- |
| **Semblance of Control** |  | Control  Drink responsibly  Know your limits | Learning from mistakes  Personal rules  Within norms |
| **Harmful Drinking Contexts** | *Mental Harms* | Depression | Guilty |
|  | *Physical Harms* | Accidents  Big groups  Big day or night out  Blackout  Gateway to other substances  Gendered concerns about harms  Getting home safe | Hangover  Long term physical health  Mugged/stolen belongings  Trouble outside pubs  Vomit  Violence  With partner |
|  | *Social Harms* | Anger  Arguing  Bothering others  Mean/nasty | Relationship damage  Rowdy behaviour  Social transgressions |
|  | *Societal Harms* | British culture  Drink driving | Social exclusion  Work drinks |
| **Features which make drinking “harmful”** | *Alone* | Drinking alone  Drinking alone is brave/ empowering  Drinking should be social  Pub with friends | Secretive  Someone looking out for you  Street drinking |
|  | *Home* | Cheap drinks  Home alone  Home is unregulated | Natural stopping point  Safe at home  Social drinking in a home |
|  | *Amount* | Contexts evolve and change  Drinking excessively | Free alcohol  Rounds |
|  | *Drink Type* | Depends on class or social status | Drink type |
|  | *Having responsibilities* | Functioning  On duty | Responsible for children |
|  | *Reason for drinking* | Alcohol as an 'aid'  Alcohol as a crutch  Alcohol isn't focus  Complimenting a meal  Celebration | Happy drinking  Helps with socialising  Pleasure  Reason  To relax |
|  | *Timing/Pattern* | Bad habits  Frequency  In the day | Morning  Repeated pattern of behaviour |

**Appendix 5 – All Images**

**Focus Group 1 – Drinking alone**


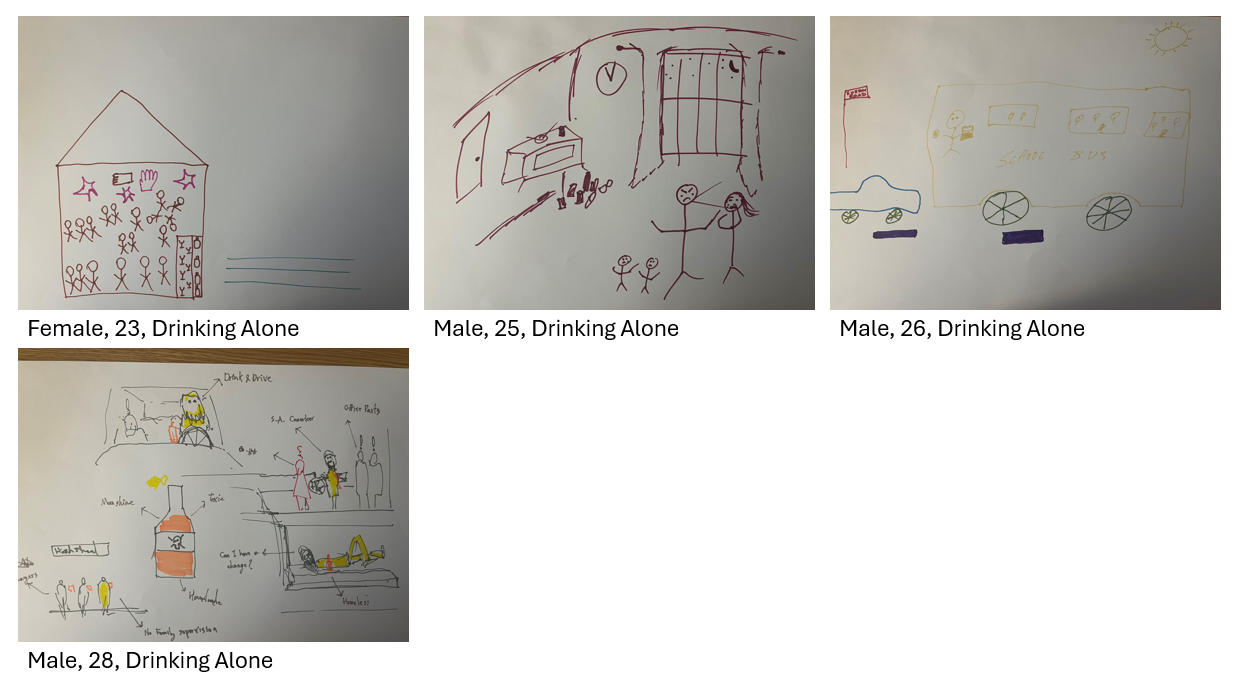


*One participant arrived late and so did not draw a picture.

**Focus group 2 – Social out of home**


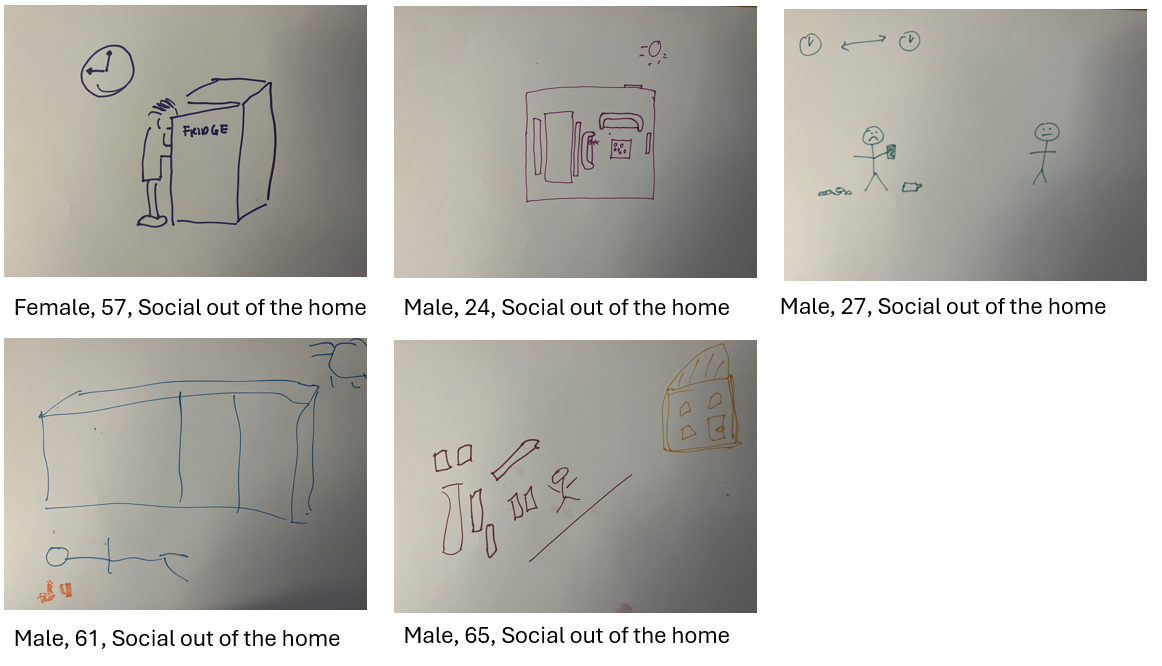


**Focus group 3 – With partner**


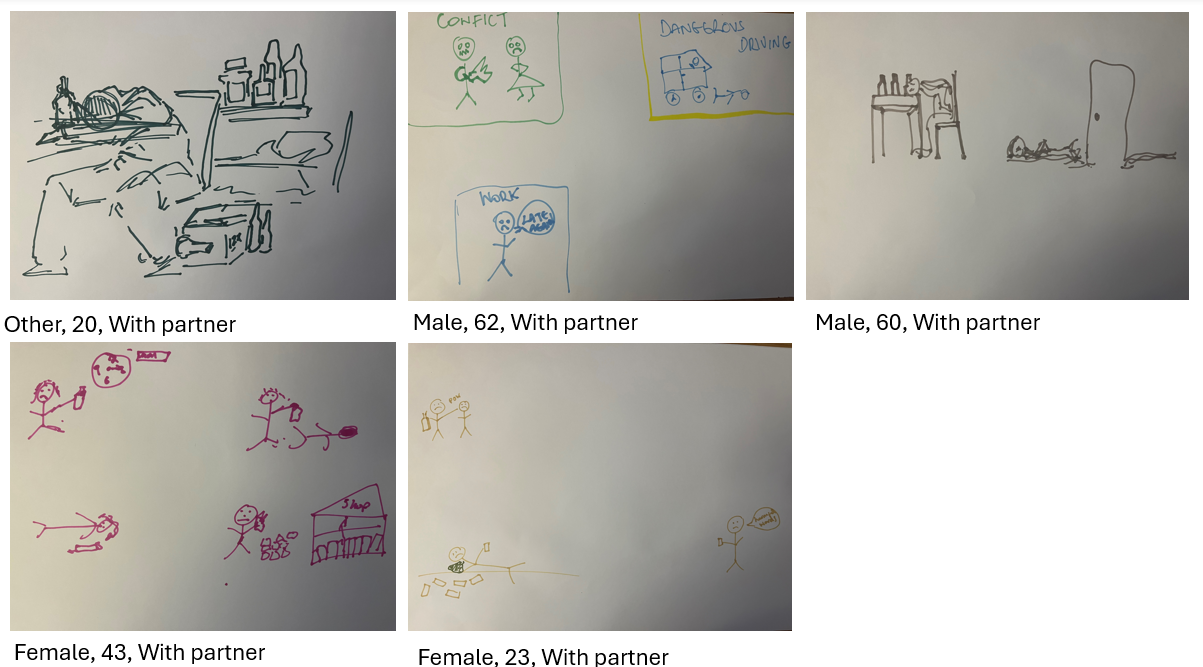


**Focus group 4 – Social in home**


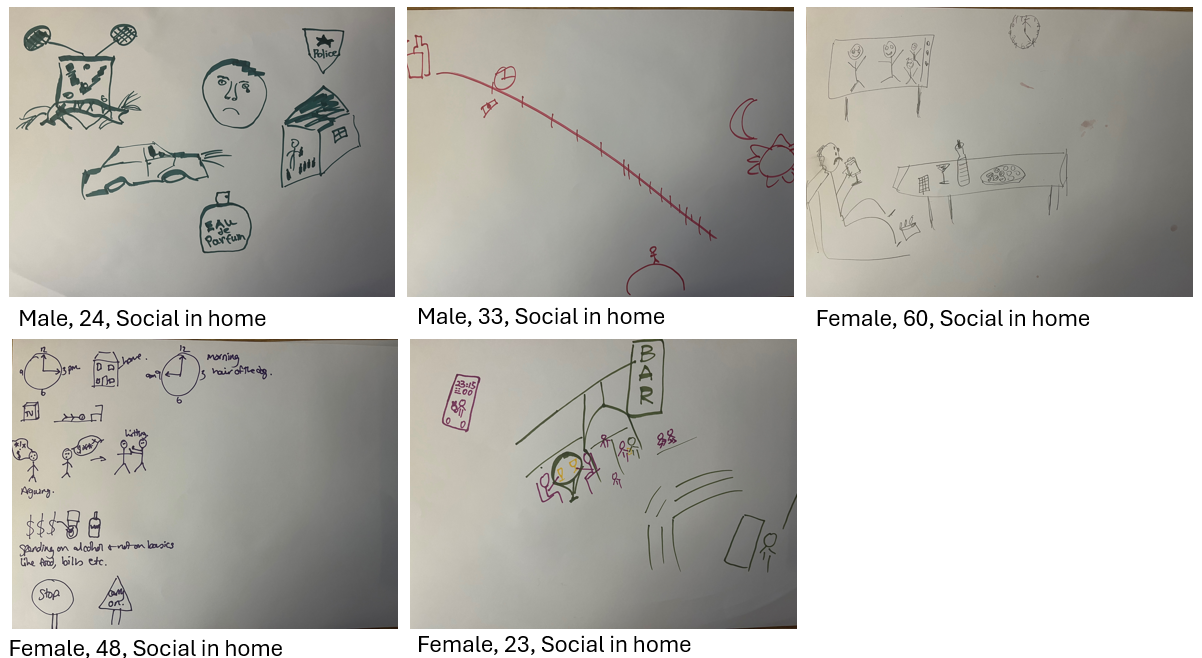

Supplement: Supplementary file 1 — Data S1: [file BJHP-30-0-s001.zip › Supplementary Materials.docx]
